# Real-Life Experience of HER2 (Human Epidermal Growth Factor Receptor 2)-Positive Advanced Breast Cancer Patients Treated With T-DXd (Trastuzumab Deruxtecan): A Multicentric Portuguese Study

**DOI:** 10.7759/cureus.79734

**Published:** 2025-02-27

**Authors:** Rita Bizarro, Isabel Pazos, Alexandra Teixeira, Margarida Pereira, Joana Gonçalves, Catarina Abreu, Rita Ferreira, Sónia Oliveira, Ana Duarte Mendes, Inês Eiriz, Mariana Santiago, Carina Teixeira, Maria Leitão, Helena Guedes, Sandra Bento, Mariana Inácio, Tiago Alpoim, Jorge Correia, Francisco Branco, José Passos-Coelho, Mafalda Casa-Nova, José Alberto Teixeira

**Affiliations:** 1 Medical Oncology, Beatriz Ângelo Hospital, Loures, PRT; 2 Medical Oncology, Francisco Gentil Portuguese Institute of Oncology Coimbra, Coimbra, PRT; 3 Medical Oncology, Senhora da Oliveira Hospital, Guimarães, PRT; 4 Medical Oncology, Francisco Gentil Portuguese Institute of Oncology Lisboa, Lisboa, PRT; 5 Medical Oncology, Nossa Senhora do Rosário Hospital, Barreiro, PRT; 6 Medical Oncology, Santa Maria Hospital, Lisbon, PRT; 7 Medical Oncology, Santo António dos Capuchos Hospital, Lisboa, PRT; 8 Medical Oncology, Prof. Dr. Fernando da Fonseca Hospital, Amadora, PRT; 9 Medical Oncology, Garcia de Orta Hospital, Almada, PRT; 10 Medical Oncology, São João Hospital, Porto, PRT; 11 Medical Oncology, São Teotónio Hospital, Viseu, PRT; 12 Medical Oncology, Vila Nova de Gaia - Espinho Hospital, Vila Nova de Gaia, PRT; 13 Medical Oncology, Santarém District Hospital, Santarém, PRT; 14 Medical Oncology, Espírito Santo Hospital, Évora, PRT; 15 Medical Oncology, Santa Luzia Hospital, Viana do Castelo, PRT; 16 Medical Oncology, Luz Hospital, Lisboa, PRT; 17 Medical Oncology, São Bernardo Hospital, Setúbal, PRT

**Keywords:** breast cancer, her-2 positive breast cancer, metastatic breast cancer, real-life evidence, trastuzumab deruxtecan

## Abstract

Background: Substantial improvements in survival have been observed in HER2 (human epidermal growth factor receptor 2)-positive (HER2+) inoperable or metastatic breast cancer (advanced breast cancer [ABC]) in recent years, driven by the introduction and widespread use of multiple novel agents. The DESTINY-Breast02 trial compared the efficacy and safety of trastuzumab deruxtecan (T-DXd) in patients with HER2+ ABC formerly treated with trastuzumab emtansine (T-DM1), demonstrating significant improvements in both overall survival (OS) and progression-free survival (PFS).

Methods: We conducted a national, multicentric, retrospective study to describe real-world treatment patterns, PFS, OS, safety, and key toxicities associated with T-DXd use in Portugal, following the DESTINY-Breast02 inclusion criteria.

Results: A total of 100 women with HER2+ ABC from 17 centers were included, all of whom had received at least two prior treatments for advanced disease and were treated with T-DXd between July 2021 and May 2023. The mean age was 53.9 years n(standard deviation: 9.9). Thirty-six patients presented with synchronous metastatic disease. The most common metastatic site was bone, in 61 (61%) patients; 72 (72%) had visceral metastases, and 21 patients (21%) had brain metastases. The median follow-up was 10 months, with a median of 11 T-DXd cycles administered. Prior treatments included pertuzumab in 71 (71%) patients and T-DM1 in 84 (84%). T-DXd was administered as third-line therapy in 52 (52%) patients, as fourth-line therapy in 15 (15%), and as fifth-line therapy and beyond in 23 (23%) patients. The overall response rate (ORR) was 44%, and the clinical benefit rate (CBR) was 80%. The most frequent toxicities of any grade were nausea in 49 patients (49%), neutropenia in 37 (37%), and alopecia in 34 (34%). Serious adverse events (grade ≥ 3) occurred in 16 (16%) patients, with treatment discontinuation or delays due to adverse events observed in 46 cases (46%). Median OS was not reached, with a 12-month OS rate of 74%. The median PFS was 13 months (95% CI: 10-16 months), and the 12-month PFS rate was 54%.

Conclusions: This real-world analysis revealed that the efficacy, safety, and tolerability of T-DXd in the Portuguese population are consistent with the outcomes observed in the DESTINY-Breast02 clinical trial.

## Introduction

Female breast cancer (BC) incidence rates have been slowly increasing by about 0.5% per year since the mid-2000s representing the most diagnosed malignancy in women. Currently, BC is the second cancer-related cause of mortality worldwide [1,2].

The human epidermal growth factor receptor 2 (HER2) is a transmembrane tyrosine kinase receptor located on chromosome 17q21. HER2 acts as an oncogene in BC, its overexpression resulting in ligand-independent dimerization that leads to constitutive activation of its cytoplasmic kinase domain. Oncogenic HER2 is overexpressed in 15%-20% of primary BC. Overexpression of HER2 in BC occurs predominantly through amplification of the HER2 gene and is associated with a more aggressive phenotype [3,4].

The transmembrane tyrosine kinase (TKI) receptor HER2 (otherwise known as ErbB2 or p185) has been proven to be an effective target therapy for patients with BC in the last 20 years [5-7].

In the advanced disease setting, the CLEOPATRA trial demonstrated superior overall survival (OS) and progression-free survival (PFS) after adding pertuzumab to trastuzumab and docetaxel in patients with HER2+ metastatic BC naïve to anti-HER2 therapy and chemotherapy for metastatic disease. After the CLEOPATRA trial, this association became the first-line standard of care for advanced HER2+ BC [5].

For a decade, trastuzumab emtansine (T-DM1) has been approved in the second-line setting. T-DM1 is an antibody-drug conjugate (ADC), which drives the chemotherapeutic agent directly to HER2-expressing cells through the driver trastuzumab [8]. Its safety and efficacy were evaluated in the EMILIA trial, in which patients with metastatic BC who received prior anti-HER2 therapy had a median PFS and OS significantly longer with T-DM1 versus lapatinib plus capecitabine [9].

Recently, the paradigm has been changed after the presentation of trastuzumab deruxtecan (T-DXd). T-DXd consists of an ADC with a humanized anti-HER2 monoclonal antibody linked to topoisomerase I inhibitor payload through a tetrapeptide-based cleavable linker [10,11].

Compared to T-DM1, T-DXd showed a higher drug-to-antibody ratio, 8 versus 3 to 4.8. In addition to direct cytotoxicity on the tumor cell, T-DXd can induce cell death in surrounding cells, an effect called the “bystander effect,” inducing cytotoxicity also on cells that do not overexpress the HER2 receptor [12].

The multicenter, phase 2, single-arm DESTINY-Breast01 study evaluated the efficacy and safety of T-DXd in HER2+ BC patients with unresectable metastatic disease who had previously received treatment with TDM-1. This study demonstrated a significant anti-tumor response to T-DXd in a population of patients with HER2+ metastatic BC who had undergone multiple lines of treatment. It was therefore approved by the FDA in December 2019 and by the European Medicines Agency (EMA) in January 2021 and is currently considered the standard treatment for patients with HER2+ metastatic BC after progression on dual HER2 blockade with a taxane and T-DM1 [10].

More recently, the phase 3 DESTINY-Breast02 study (DB02), which compared the efficacy of T-DXd versus investigator’s choice therapy in patients with metastatic or unresectable HER2+ BC, after T-DM1, showed statistically significant results for PFS and OS in the T-DXd treatment group [13].

The phase 3 DESTINY-Breast03 study (DB03), which included patients who had progressed on taxane and dual HER2 blockade with trastuzumab and pertuzumab, showed significant gains in PFS and OS in patients treated with T-DXd compared to T-DM1. Currently, T-DXd is considered the standard second-line therapy in patients previously treated with one or more anti-HER2 target regimens [14,15].

In Portugal, the INFARMED (National Authority of Medicines and Health Products) has approved the reimbursement for T-DXd after one or more previous anti-HER2 target therapies in patients with unresectable or metastatic HER2+ BC.

Although improved efficacy is important, toxicity and its effect on quality of life (QoL) are significant and equally important to patients. Pulmonary and cardiovascular toxicity, especially interstitial lung disease (ILD), is a toxicity of concern with some ADCs including T-DXd that appear to be dose- and frequency-dependent. Fatigue, hematological, and gastrointestinal toxicities have also been found in a significant proportion of patients. Therapeutic toxicities were also described in this study.

In this study, we aimed to evaluate the real-world efficacy and safety of T-DXd in patients with HER2+ advanced BC in Portugal, using PFS, OS, and toxicity profiles as primary endpoints. Secondary objectives include assessing treatment patterns and comparing real-world outcomes to findings from the DESTINY-Breast02 trial. To our knowledge, there are no previous similar studies with T-DXd in the Portuguese population.

The results of this study were previously presented as a meeting abstract and poster at the ESMO (European Society for Medical Oncology) Breast Cancer 2024 Annual Congress, which took place in Berlin, Germany, on 15th-17th May 2024.

## Materials and methods

Patient population and enrollment criteria

This is a retrospective, multi-institutional study from 17 Portuguese medical centers that included patients with HER2+ metastatic BC treated in Portugal, with at least one administration of T-DXd for advanced disease. Eligible patients were identified from electronic medical records using predefined criteria. Selection was performed at each center under standardized inclusion protocols. Patients with less than six months of treatment were excluded. There was no limit for previous lines of treatment. HER2 positivity was tested at each center and defined as 3+ immunohistochemical (IHC) staining or 2+ IHC staining and positive fluorescence in situ hybridization test (FISH) or silver-enhanced in situ hybridization (SISH) [16]. Demographic and clinicopathological data from the computerized medical records of the included patients were analyzed. All data were collected with pseudonymization.

Study objectives and endpoints

After the characterization of the population with HER2+ BC (clinical and demographic variables) treated with at least two prior treatments of T-DXd in Portugal, the primary endpoints were OS and PFS. The secondary endpoints were overall response rate (ORR) and safety. Baseline demographic characteristics included age, Eastern Cooperative Oncology Group (ECOG) performance status, site and number of organs with metastases, and previous lines of treatment in the metastatic setting. RECIST v1.1 (response evaluation criteria in solid tumors) criteria were applied locally to evaluate the radiological response and disease progression. Imaging intervals were decided by the treating physicians, which were mostly based on clinical judgment and institutional practice as well as decisions on treatment discontinuation or dose adjustments. OS was defined as the time from the start of T-DXd treatment to death due to cancer or any cause. PFS was defined as the time from the start of treatment to evidence of disease progression or death. ORR was defined as complete and partial responses. Clinical benefit rate (CBR) was defined as partial response (PR), complete response (CR), or stable disease (SD). Adverse events (AEs) were graded based on the Common Terminology Criteria for Adverse Events (CTCAE), version 5.0 [17,18].

Statistical analysis

All statistics were performed using the SPSS software v28.0 (IBM Corp., Armonk, NY). The Kaplan-Meier method was used to estimate survival outcomes (PFS and OS) and conducted with right-censored data.

## Results

Study population

Between July 2021 to May 2023, 100 patients from 17 Portuguese centers were enrolled. The mean age was 53.9 years (standard deviation: 9.9). Of the 100 patients, 86 (86%) were age 65 years old or younger, while 14 (14%) were older than 65 years. All patients included were women. The median ECOG PS (performance status) score was 1 (range: 0-2). Sixty-nine (69%) patients had hormone receptor-positive/HER2+ (HR+/HER2+) disease on primary site tumor, and the remaining 31 (31%) patients had HR-negative/HER2+ (HR−/HER2+) BC.

The most frequent histological type of BC was the no special type (NST) in 93 (93%) patients. The most common metastatic sites were lymph nodes and bone, in 69 (69%) and 61 (61%), respectively. The majority, 71 individuals (71%), had received pertuzumab, and 84 (84%) had received T-DM1 previously. The median duration of treatment in the previous line was seven months (range: 2-68 months).

For metastatic disease, the median number of previous therapeutic lines was 2 (range: 1-7). The patient characteristics are reported in Table 1.

**Table 1:** Baseline characteristics and previous treatments N(%): Number of patients (percentage); HER2: Human epithelial receptor 2; ICH: Immunohistochemistry; ISH: In situ hybridization; NST: Invasive ductal carcinoma of no special type; T-DM1: Trastuzumab emtansine; T-DXd: Trastuzumab deruxtecan.

Baseline Characteristics and Previous Treatments	
Variables	Value, N (%)
*Sex, N (%)*	
Female	100 (100.0)
*ECOG PS, N (%)*	
0	48 (48.0)
1	45 (45.0)
2	6 (6.0)
Unknown	1 (1.0)
*Age at the start of T-DXd, N (%)*	
≤65 years	86 (86.0)
>65 years	14 (14.0)
*Hormone receptor, N (%)*	
Positive	69 (69.0)
Negative	31 (31.0)
*HER2 status on primary tumor, N (%)*	
ICH 3+	78 (78.0)
ICH 2+, ISH-positive	22 (22.0)
*HER2 status on available metastases, N (%)*	
ICH 3+	40 (78.4)
ICH 2+, ISH-positive	11 (21.6)
*Histological type, N (%)*	
NST	95 (95.0)
Lobular	2 (2.0)
Papillary	3 (3.0)
*Previous anti-HER2 treatments, N (%)*	
Pertuzumab	71 (71.0)
T-DM1	84 (84.0)
*Time to progression after adjuvant anti-HER2 treatment, N (%)*	
<6 months after adjuvant trastuzumab	12 (12.0)
Metastatic ab initio	36 (36.0)
*Metastatic disease location, N (%)*	
Lymph nodes	69 (69.0)
Bone	61 (61.0)
Liver	56 (56.0)
Lung	54 (54.0)
Brain/spinal cord	21 (21.0)
Received local therapy	14 (14.0)
*Other sites*	
Skin	21 (21.0)
Pleura	5 (5.0)
Ovary	4 (4.0)
Adrenal gland	2 (2.0)
Peritoneum	2 (2.0)
Contralateral breast	1 (1.0)
*Number of previous lines of therapy for advanced disease for metastatic disease, N (%)*	
1	10 (10.0)
2	52 (52.0)
3	15 (15.0)
4	6 (6.0)
5	6 (6.0)
6	6 (6.0)
7	5 (5.0)
Median (range)	2 (1-7)

Outcomes

Among the study population, patients received T-DXd (5.4 mg per kilogram of body weight) every three weeks, and the treatment was continued until progression or unacceptable toxicity. The median follow-up was 10 months with 11 median cycles of T-DXd administered (range: 1-33).

**Figure 1:**
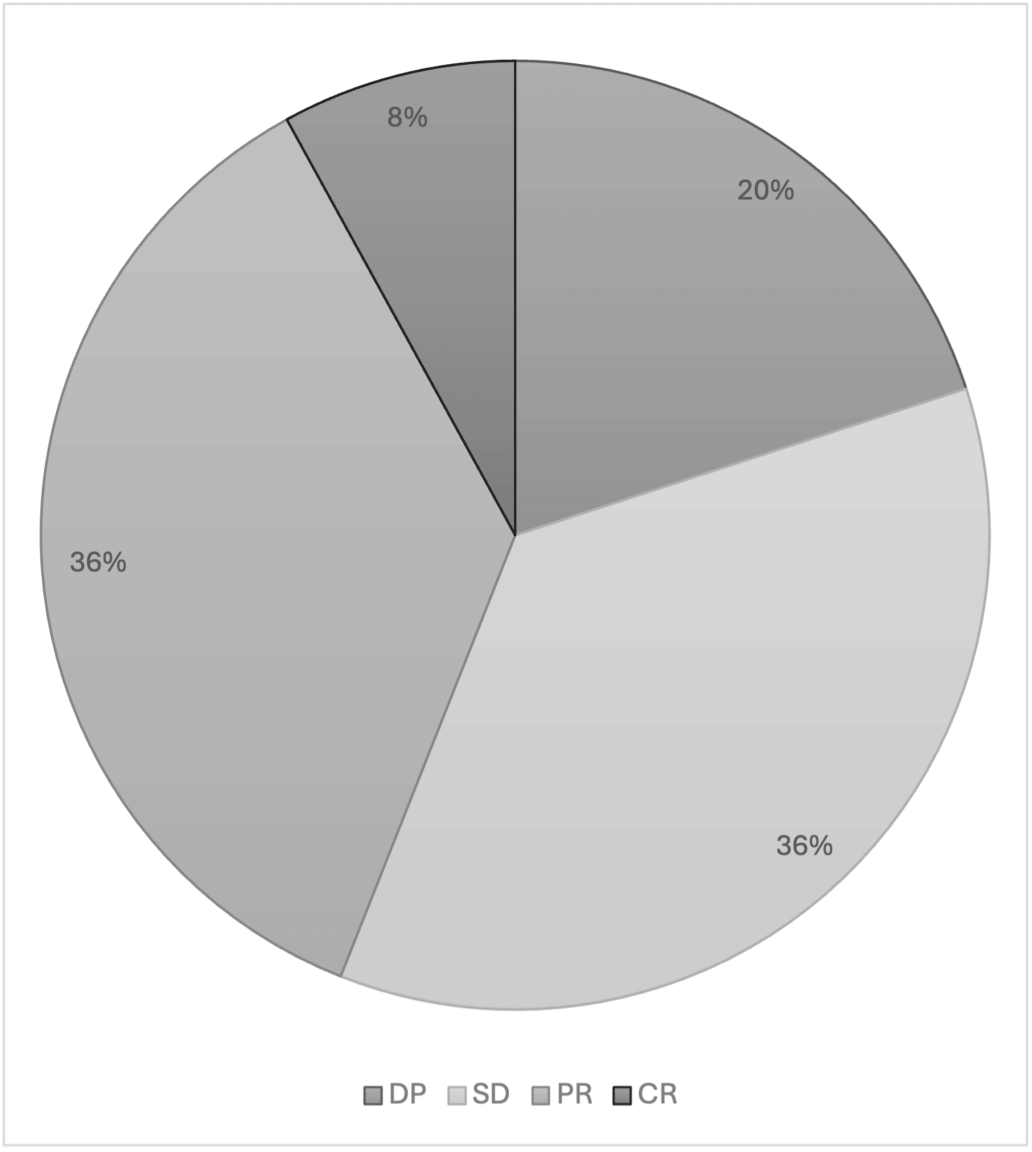
Best response to T-DXd CR: Complete response; PR: Partial response; SD: Stable disease; DP: Disease progression; T-DXd: Trastuzumab deruxtecan.

The ORR was 44%, and the CBR was 80%. Among these patients, eight (8%) had a CR, 36 (36%) had a PR, and 36 (36%) reported a stable disease (SD). In 20 patients (20%), a primary resistance to T-DXd was detected. Figure 1 and Tables 2, 3 illustrate the best responses to T-DXd observed in the study population.

**Table 2:** Best response to T-DXd N (%): Number of patients (percentage); CR: Complete response; PR: Partial response; SD: Stable disease; DP: Disease progression; T-DXd: Trastuzumab deruxtecan.

Best Response	Patients, N (%)
DP	20 (20.0%)
SD	36 (36.0%)
PR	36 (36.0%)
CR	8 (8.0%)

**Table 3:** Response outcomes in percentages ORR: Overall response rate; CBR: Clinical benefit rate.

Response Outcomes	
ORR	44.0%
CBR	80.0%

**Figure 2:**
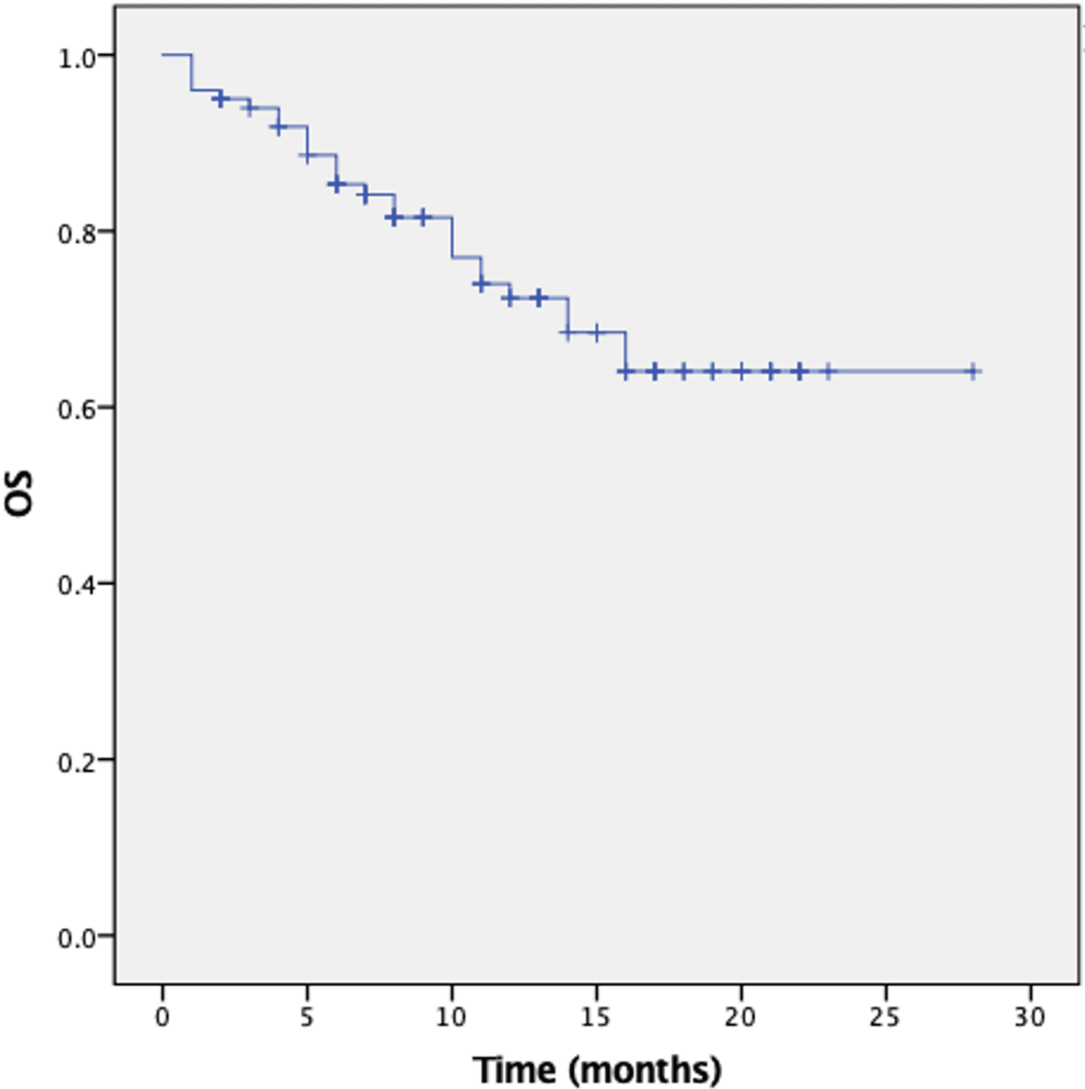
Overall survival in population treated with T-DXd. This image presents the Kaplan-Meier estimates of OS. T-DXd: Trastuzumab deruxtecan; OS: Overall survival.

**Figure 3:**
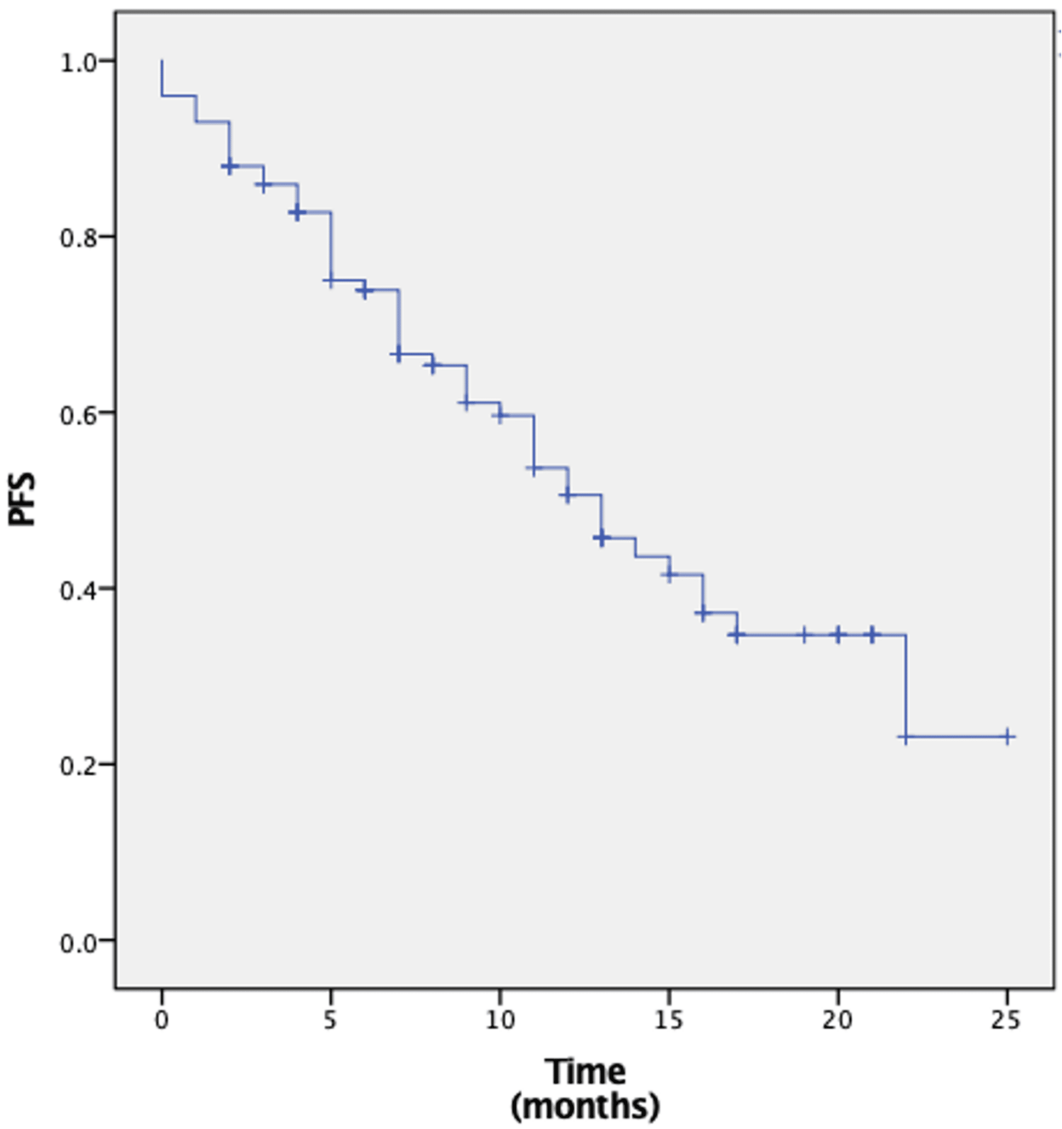
PFS in overall population treated with T-DXd. This image presents the Kaplan-Meier estimates of PFS. T-DXd: Trastuzumab deruxtecan; PFS: Progression-free survival.

Regarding survival outcomes, the median OS was not reached. OS rate at 12 months was 74% (Figure 2). The median PFS was 13 months (95% CI: 10-16 months). The PFS rate at 12 months was 54% (Figure 3).

Safety

**Figure 4:**
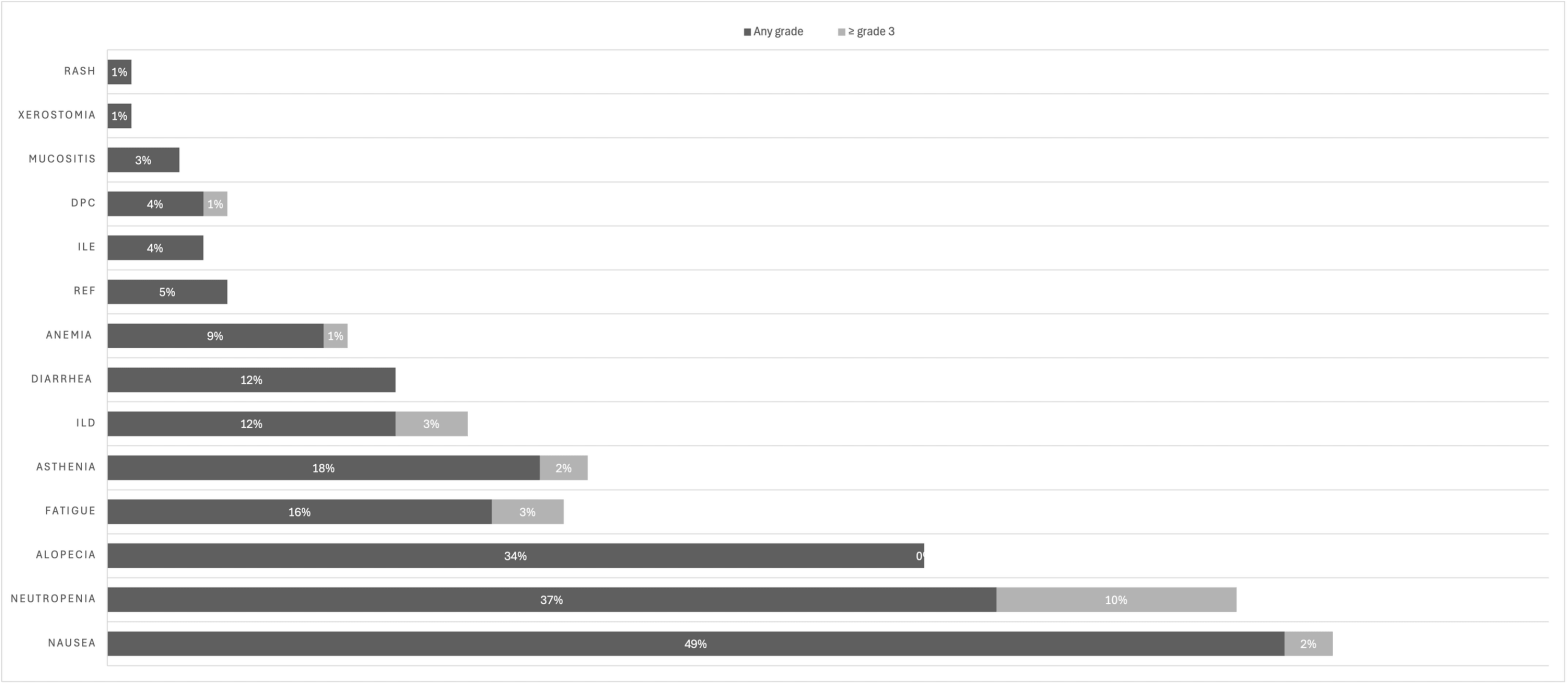
Rate of overall AEs and grade 3 AEs according to NCI-CTCAE DPC: Decreased platelet count; ILE: Increased liver enzymes; REF: Reduced ejection fraction; AEs: Adverse events; NCI: National Cancer Institute; CTCAE: Common Terminology Criteria for Adverse Events.

AEs of any grade were present in 83 individuals. The most frequent toxicities were nausea in 49 patients (49%), neutropenia in 37 (37%), and alopecia in 34 (34%). Grade 3 or higher AEs were present in 16 patients, 10 (10%) had neutropenia, three (3%) had fatigue, and three (3%) had ILD. Reduced ejection fraction (REF) of any grade was seen in 5% of patients. Dose reduction or treatment discontinuation due to AEs was required in 46 patients (46%). Regarding ILD of any grade, seven (58%) needed oral corticotherapy, and five (42%) needed intravenous (IV) corticotherapy. One patient worsened even with invasive mechanical ventilation (IMV), which led to one grade 5 drug-related death event due to ILD. The rate of overall AEs and grade 3 AEs according to CTCAE is documented in Figure 4.

## Discussion

This real-world report aimed to evaluate the efficacy and safety of T-DXd in a Portuguese patient population and compare it with the results of randomized controlled trials (RCTs).

In comparison with the DB02 trial, our study population had a similar mean age (53.9 vs 54.2 years) [13]. We reported ECOG PS of 0, 1, and 2 in 45 (45%), 48 (48%), and 6 (6%) patients, respectively, in our study versus 228 (56%), 177 (44%), and 1 (<1%), respectively, in DB02 trial [13]. Regarding population characteristics, hormonal receptor positivity was found in 69 individuals (69%) of our cohort in comparison with 238 (59%) of those in DB02 [13]. Considering HER2 status, we found HER2 3+ in 78 patients (78%) and HER2 2+ ISH-positive in 22 (22%) compared with 326 (80%) and 79 (19%), respectively, on DB02 [13]. Of note, among our patients, 71 (71%) received pertuzumab, and 84 (84%) received T-DM1 previously. On DB02, 318 patients (78%) had received pertuzumab, while almost the total population, 404 (>99%), had T-DM1 before T-DXd. In more detail, we reported slightly lower one-year PFS (54%) and OS (74%) compared to DB02 (63% and 89%, respectively) [12,13,19].

In the DB02 trial, patients were treated with a median of two prior lines before receiving T-DXd, similar to our population [13]. Compared to our data, we found a more heavily pretreated population (fourth line and beyond of 23% versus 17% on DB02). The administration of T-DXd in later lines may have explained the slightly lower ORR in our sample compared to the DB02 trial (44% vs 70%). Concerning ORR, we reported 44% versus 70% in DB02. Considering this difference, we believe that the heavily pretreated population experienced reduced objective responses, as T-DXd was administered in subsequent lines. Other causes could be real-world variations in supportive care, toxicity management, or adherence [2,13,19].

Emesis, neutropenia, alopecia, and fatigue were the most frequently reported clinically relevant AEs of any grade and were found in 83 (83%) patients. We noted nausea in 49 (49%) patients, neutropenia in 37 (37%), alopecia in 34 (34%), fatigue in 16 (16%), asthenia in 18 (18%), ILD in 12 (12%), and diarrhea in 12 (12%) of our population. In DB02, the main toxicities were nausea in 73% of patients, alopecia in 37%, fatigue in 36%, diarrhea in 27%, and ILD in 10%. In our population, grade 3 or higher AEs were found in 16 (16%) versus 53% in the DB02 trial. Dose reductions and treatment discontinuation were required in 46 (46%) patients owing to any AEs. Grade 3 or higher AEs reported were neutropenia in 10 patients (10%), alopecia in three (3%), fatigue in three (3%), and ILD in three (3%) compared to anemia (8%), neutropenia (8%) and nausea (7%) in the DB02 trial [13]. In this trial, 24% of patients required dose reductions, and 18% had treatment discontinuation.

A limitation of this study is that in real-world analysis, due to less stringent protocols, clinicians may not record toxicities as thoroughly, which may lead to an underestimation of AEs. Other additional limitations of our study are the small sample size and the heterogeneity in the timing of imaging assessment among the different centers. The retrospective design of this study and the potential for selection bias could be other limitations [20,21].

## Conclusions

In our analysis, it was observed that T-DXd is safe, active, and effective in a real-world population, with comparable results to RCTs. The relatively small sample size may limit statistical power, potentially leading to an underestimation or overestimation of treatment effects. To give more strength to these findings, a study with a larger population and an extended follow-up would be important to corroborate the efficacy and safety of this medication among patients with HER2+ ABC. The study does not include adjustment for confounding factors such as prior therapy exposure, which may have influenced treatment response. The lower ORR observed in our study compared to DB02 could be influenced by multiple factors, including differences in patient selection, prior treatment regimens, or variations in toxicity management in real-world settings. Our findings on T-DXd-related toxicities align with clinical trial data; however, future studies should compare these outcomes with other real-world datasets to further validate safety findings. Real-world studies are important to provide evidence to clinicians and give essential information about the safety and effectiveness of new therapeutics.
